# Ureteral stricture: current treatment algorithm and key surgical principles in the robotic upper urinary tract reconstruction era

**DOI:** 10.1007/s00345-025-06181-4

**Published:** 2026-01-17

**Authors:** Alice Bourillon, Benoit Peyronnet, Barry B. McGuire, Ugo Pinar, Ziho Lee, Rajesh Nair, Michael Stifelman, Daniel Eun, Lee C. Zhao, Nikita Bhatt

**Affiliations:** 1 Department of Urology, University Hospital of Rennes, 2 Rue Henri Le Guilloux, Rennes, France; 2 Department of Urology, St Vincent’s University Hospital, Dublin, Ireland; 3 Department of Urology, La Pitié Salpêtrière Hospital, Sorbonne University, Paris, France; 4 Department of Urology, Northwestern University Feinberg School of Medicine, Chicago, IL USA; 5 Department of Urology, Guy’s Hospital, London, UK; 6 Department of Urology, Hackensack Meridian School of Medicine, Nutley, NJ USA; 7 Department of Urology, Lewis Katz School of Medicine at Temple University, Philadelphia, PA USA; 8 Department of Urology, NYU Langone Health, New York, USA

**Keywords:** Robotics, Ureter, Stricture, Reconstruction, Treatment

## Abstract

**Purpose:**

Robot-assisted surgical management of ureteral stricture is a relatively uncommon procedure with numerous techniques described. This literature review combined with expert opinion outlines the key surgical principles of ureteral reconstruction and proposes an algorithm for the robot-assisted treatment of ureteral strictures.

**Methods:**

A narrative literature review was conducted using PubMed and Cochrane databases with a predefined search strategy including terms related to ureteroplasty and upper urinary tract reconstruction. The search yielded approximately 900 results. After screening, 219 relevant studies were selected for analysis. Two authors independently reviewed all included articles, and whenever evidence was inconsistent or insufficient, consensus was reached through discussion within the full author group.

**Results:**

Robotic ureteral reconstruction includes several techniques, with the choice of procedure guided by multiple factors. Key principles include the use of healthy tissue, creation of a tension-free anastomosis, and ensuring adequate drainage. Outcomes may be further optimized by preoperative ureteral rest, intraoperative ureteroscopy, and the use of intravenous indocyanine green (ICG). Buccal mucosa grafting has emerged as a breakthrough in minimally invasive reconstruction. The robotic approach is adept at managing standard techniques such as ureteroureterostomy, psoas hitch, Boari flap, ureteroneocystostomy, and more recently described methods such as non-transecting ureteral reimplantation and bladder flap ureteroplasty. Ileal ureter remains a good option in cases of challenging reconstructions and patients with prior radiation therapy or extensive defects.

**Conclusion:**

Robot- assisted ureteral reconstruction has shifted the paradigm in stricture management, allowing complex reconstructive operations to be performed with minimally invasive techniques. This expert review summarizes key surgical approaches and presents a practical algorithm to guide urologists in the management of ureteral strictures.

## Introduction

The treatment of ureteral stricture diseases is complex and nuanced, and it currently lacks consensus [1]. Due to the lack of a universally accepted threshold of pathological ureteral lumen narrowing, the definition of ureteral stricture remains uncertain [2].

Familiarity with ureteral stricture management is vital for urologists as one is bound to encounter it during their practice. Managing ureteral strictures with regular stent changes is a suboptimal treatment option which can significantly affect the patient’s quality of life. It should be reserved for palliative cases [3, 4].

From the first open appendicoplasty for ureteral repair in 1912, surgical techniques and approaches have continuously evolved, shaping new paradigms in upper urinary tract reconstruction. Current practice of management of ureteric strictures has been revolutionized by the application of robotic upper urinary tract reconstruction and use if buccal mucosa graft ureteroplasty. However, the evidence in this area remains scarce, heterogenous and of poor quality with small patient numbers. There is a lack of guidelines in the management of ureteric stricture disease resulting in ambiguity in patient management.

The aim of this review is to summarize the current knowledge on robotic ureteral reconstruction (RUR), to outline the key surgical principles and to propose an algorithm to guide clinical decision making.

## Material and methods

Experts in the field of robotic ureteral reconstruction (RUR) were identified based on their published work and their participation as faculty in relevant surgical courses.

Clinical questions regarding RUR were formulated by the authors and circulated among the panel of experts to identify the issues most pertinent to practice.

A literature review was conducted using PubMed and Cochrane, applying a search algorithm (Appendix 1) with key terms related to ureteroplasty and upper urinary tract reconstruction. The evidence obtained was used to answer the important clinical questions identified during the initial phase and discussed among the panel of experts to ensure it was acceptable. In cases where the evidence was insufficient, expert opinion was used to answer the question.

This work represents a narrative literature review supplemented by expert consensus rather than a systematic review; therefore, a formal risk of bias assessment was not conducted.

The themes discussed in the review include indications for surgery including various available techniques for each indication, key surgical principles of RUR, and the perioperative management. We further provide an expert-derived algorithm to guide clinical practice using the evidence and expert opinion of this panel.

## Results

The literature search yielded approximately 900 results, including many pediatric cases. After screening, 219 relevant studies were selected for analysis (Annex 1: Search Algorithm).

### What is the role of endoscopic treatment in the era of robotic upper tract reconstruction?

The success rate of endoscopic management of benign ureteral strictures is approximately over 50% at 1 year, and higher in strictures measuring < 2 cm compared with those ≥ 2 cm, with an odds ratio of 0.13 in favor of shorter strictures. However, the evidence underpinning this is weak [5]. Longer strictures, strictures of iatrogenic cause and stricture in radiated patients have lower success rates [6]. Hence, while for most simple strictures, endoscopic treatment—preferably balloon dilation—can be considered as a first-line approach, it should be discussed with the patient as part of a shared decision-making model of care. Due to the higher rate of failure, repeated endoscopic treatments should be avoided. Endoscopic management has low success for complex strictures, and upfront surgical management should be offered.

Based on the evidence available and the panel’s opinion, endoscopic treatment should be reserved for simple ureteral strictures (< 2 cm, non occlusive) as initial attempt, repeated attempts at treatment should be avoided and the minimum possible duration of stenting should be used prior to considering definitive repair, unless it is a palliative treatment option.

### What are the advantages of a robotic approach for ureteral reconstruction?

The robotic platform matches all the ability of open surgery enabling precise tissue dissection and preservation of vascular supply, which are critical in ureteral surgery. However, the surgery can be delivered minimally invasively even when multiquadrant areas are required (which in open surgery either meant large or multiple incisions). Ureteral surgery can be complex and demands a high degree of adaptability from the surgeon. The planned reconstructive approach may need to be modified intraoperatively, as the true extent and nature of the stricture often only becomes fully apparent only during the procedure. Consequently, it is crucial to ensure that patients are thoroughly prepared and well-informed about the range of possible techniques and their associated outcomes. Robotic surgery provides the flexibility needed to adapt intraoperatively to different reconstructive techniques without significantly increasing postoperative morbidity or resulting in unfavorable cosmetic outcomes. It also allows for the use of indocyanine green (ICG), a valuable adjunct in ureteral reconstruction that enhances tissue visualization beyond what is possible with the naked eye. Experimental studies suggest that robotic dissection may lead to reduced fibrotic periureteral tissue formation compared to open surgery. It can theorized that the robotic approach may be more favorable when redo procedures are necessary in the future due to reduced risk of adhesion formation [7].

Although results remain debated, the robotic approach has advantages over the laparoscopic approach in terms of shorter operative times and length of stay across various procedures—such as ureteroureterostomy, ureteroneocystostomy, and laparoscopic-assisted mucosal grafting (LMG)—albeit with higher associated costs [8–11]. Some studies report longer operative times, likely reflecting the learning curve and the surgeon’s level of experience [12–14]. However, the main advantage the robotic approach has over standard laparoscopy is the dexterity and freedom of movement with challenging angles, dissection and suturing, not to mention less attrition to the surgeon due to superior ergonomics.

The robotic approach outperforms open surgery in many techniques, such as ileal ureter ureteroneocystostomy, and BMG, offering shorter hospital stays, reduced Foley catheterization and JJ stent duration, as well as reduced blood loss [15, 16].

Finally, training with the robotic approach is more straightforward with high reproducibility and shorter learning curve. The dual console, the possibility to videorecord the procedures, and the simulator are major assets in its generalization.

Overall, based on the current evidence and the panels’ experience, a robotic approach is favored for ureteral reconstruction.

### Key surgical principles

**Fig. 1:**
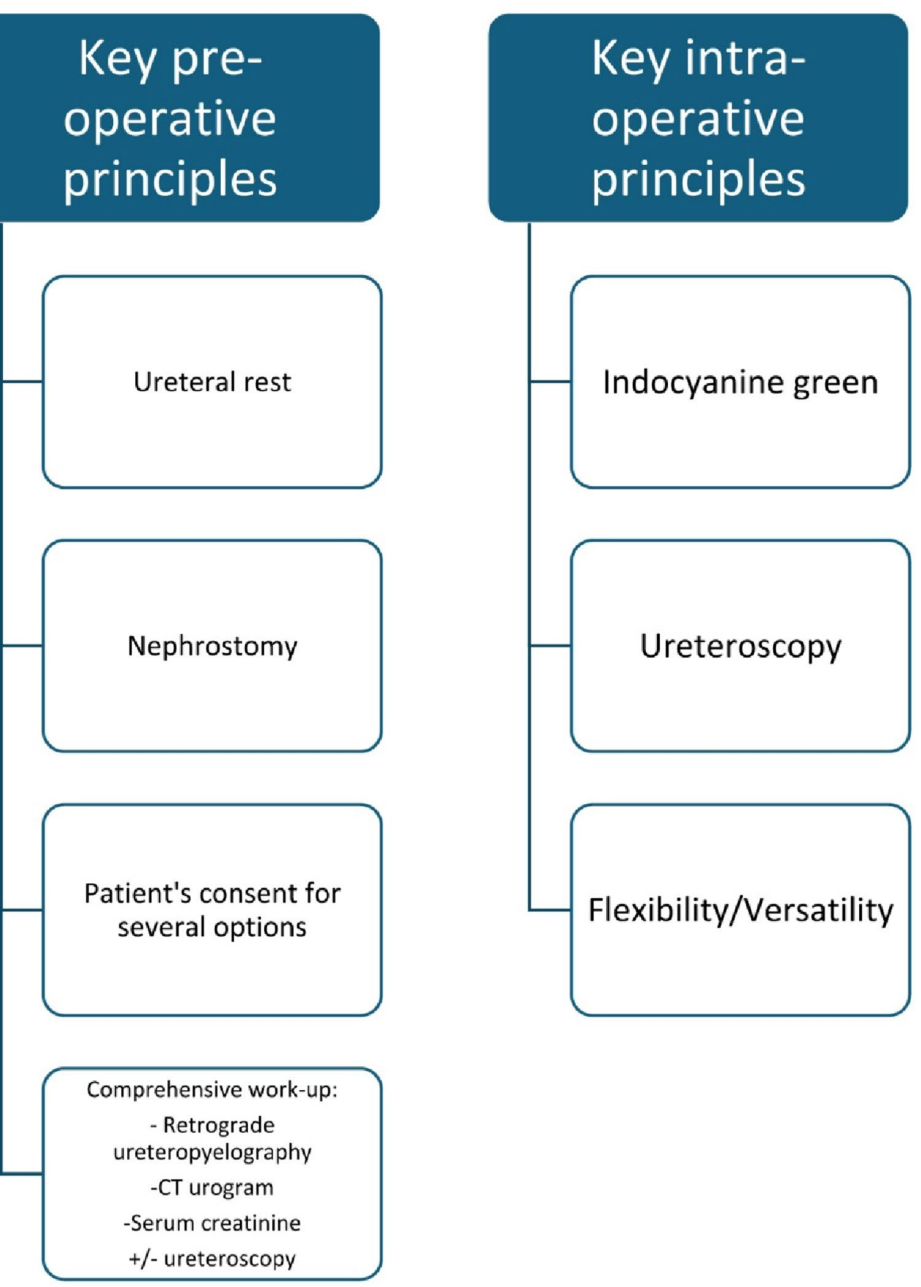
Key principles in ureteral reconstruction

The key surgical principles are outlined in Fig. 1.

#### Patient’s positioning and ports placement

**Fig. 2:**
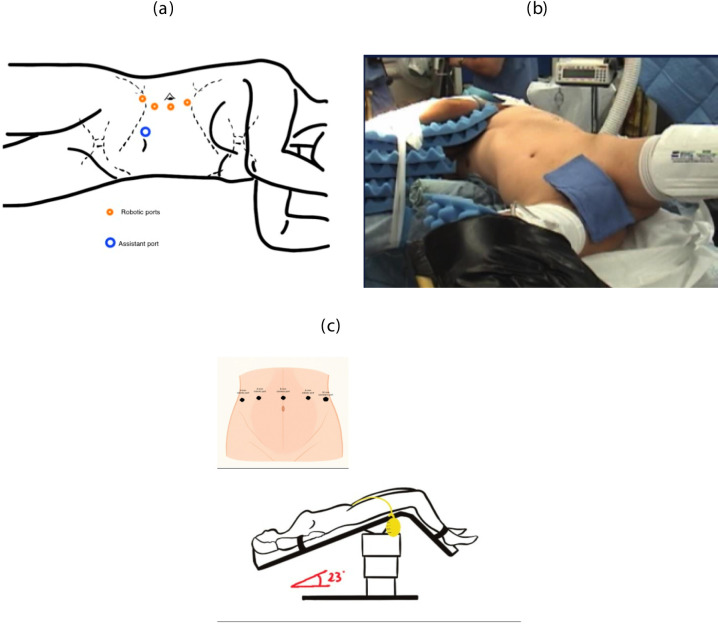
Ports’ placement and patient’s positioning. **a** Patient in a flank position, with ports placed on a straight line along the rectus muscle and the assistant port on the midline, as for a standard robotic kidney surgery. **b** Egyptian position. The patient is in Trendelenburg with spread legs to access the urethra, with the upper part of the body turned on the right to access the nephrostomy. **c** Patient in a Trendelenburg position, with spread legs and a standard port placement for robotic pelvic procedure. The camera port is on midline, a couple of centimeters above the umbilicus

The patient positioning and port placements are heavily dependent on the location of the stricture (Fig. 2). The patient is usually placed in a flank position for proximal ureteral strictures and in a 20° to 30° Trendelenburg with spread legs for iliac and pelvic ureter strictures (Fig. 2a and 2c). In the latter case, the patient’s shoulder can be turned to access the nephrostomy on the side of the stricture (Egyptian position, Fig. 2b). Four robotic ports are used according to the standard set-up for robotic proximal ureteral and robotic pelvic surgery. One 12-mm assistant port is used and an extra 5-mm assistant port is added on a case-by-case basis. This surgical set-up is used with the Intuitive DaVinci Si, X and Xi system but with newer robotic platforms the port positioning may differ. A retroperitoneal approach can also be used with different ports placement. For single port, a 3-cm incision is made between the anterior superior iliac spine and the umbilicus (low anterior supine access). An additional 8-mm assistant port can be added if necessary[17, 18].

### Indocyanine green (ICG)

**Fig. 3:**
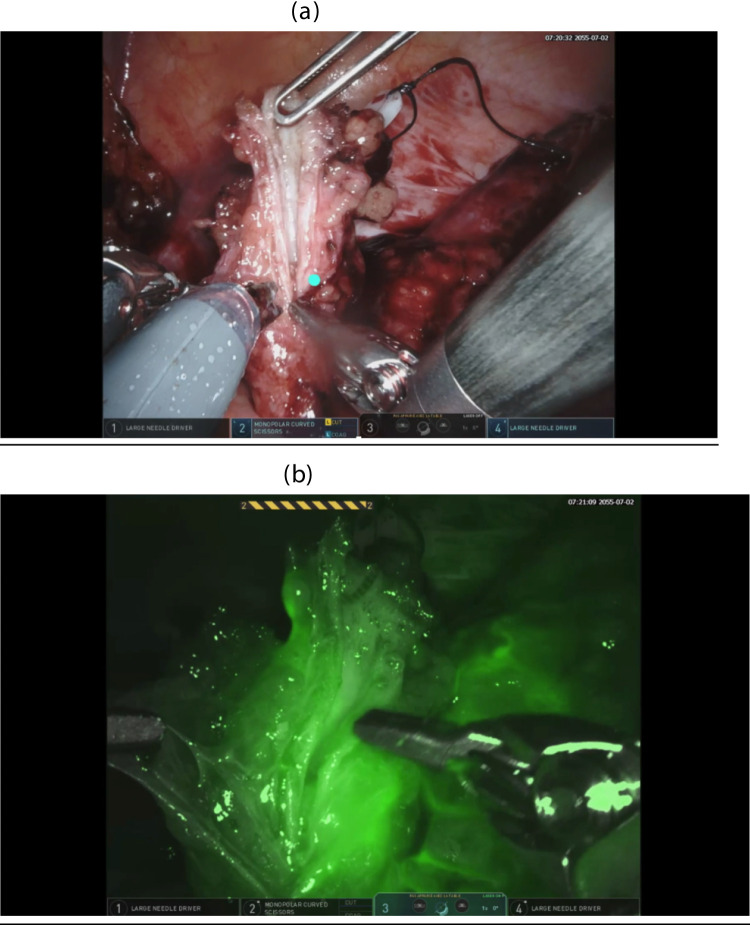
Visualization of ureteral vascularization using intravenous ICG. **a**: The vascularization of the tip of the ureter is doubtful. **b** Intravenous ICG confirm that the tip of the ureter is not well-vascularized and should be excised to ensure a successful anastomosis

Indocyanine green (ICG) is an injectable fluorescent dye which demonstrates vascularization when visualized with a robotic near-infrared camera. Its use in ureteral reconstruction has become an important adjunct in complex cases, though it is not required in every procedure. ICG offers several practical applications that can guide intraoperative decision-making:

(i) Assessment of vascularity—Intravenous injection of ICG can help identify well-vascularized, healthy portions of the ureter, thereby influencing the choice of anastomotic site and avoiding devascularized segments (Fig. 3a, b).
(ii) Ureteral localization—ICG can be injected via a nephrostomy tube, ureteroscope, or ureteric catheter to facilitate identification of the ureter in cases in patients with adhesions from prior surgery.
(iii) Reducing ischemic strictures—In uretero-ileal reconstructions, ICG can assist in ensuring that the anastomosis is performed within a well-vascularized zone, thereby minimizing the risk of ischemic stricture formation [19].

ICG is considered safe, with very few reported adverse outcomes. Potential complications are rare but may include mild allergic reactions, hypotension, or, very rarely, anaphylaxis [20]. A recent review of the literature reported favorable outcomes with ICG use in preventing ureteral stricture recurrence, with minimal complications [21]. Furthermore, some authors advocate for its prophylactic use in gynecological and colorectal surgeries to reduce the risk of iatrogenic ureteral injury [22, 23].

The expert panel supports the use of intravenous indocyanine green is an important tool for RUR.

### Ureteral rest

Ureteral rest has become a fundamental principle in ureteral reconstruction, with its benefits increasingly supported by recent studies [24, 25]. The rationale is that a period without a stent allows reduction of inflammation and oedema—factors that make surgery technically more challenging. In addition, rest facilitates clearer intraoperative visualization of the stricture and surrounding tissue.

A key advantage is that the absence of a stent helps define the true limits of the stricture, reducing the risk of undertreatment and residual fibrosis that may predispose to recurrence. Anecdotally, some surgeons also note that ureteral rest renders the ureter more supple and easier to dissect.

Approaches to ureteral rest vary. Some surgeons remove the stent 1–6 weeks before surgery; others routinely exchange it for a nephrostomy tube. Another strategy is to remove the stent and rely on antibiotics and analgesia until surgery, inserting a nephrostomy only if symptoms become intolerable. Placement of a nephrostomy tube offers the additional advantage of providing antegrade access for intraoperative ureteroscopy, leak testing any anastomosis and providing a safety valve for anastomotic rest to prevent urine leak.

Evidence suggests that ureteral rest significantly reduces the risk of stricture recurrence. Two large multicenter series demonstrated a decrease in recurrence rates from 13.4 to 4.6% and from 22.5 to 9.3%, respectively [24, 25]. Beyond stricture recurrence, ureteral rest has also been shown to improve tissue healing and reduce perioperative bleeding [24].

The optimal duration of ureteral rest remains controversial, with reported intervals ranging from as short as two days to as long as eight weeks in the literature.

Overall, the panel recommends 1–6 weeks of ureteral rest prior to attempting definitive repair of the stricture.

### Intraoperative ureteroscopy

The authors consider intraoperative ureteroscopy to be an essential adjunct in robotic ureteral reconstruction. Its routine use provides multiple benefits that enhance both surgical precision and safety.

**Fig. 4:**
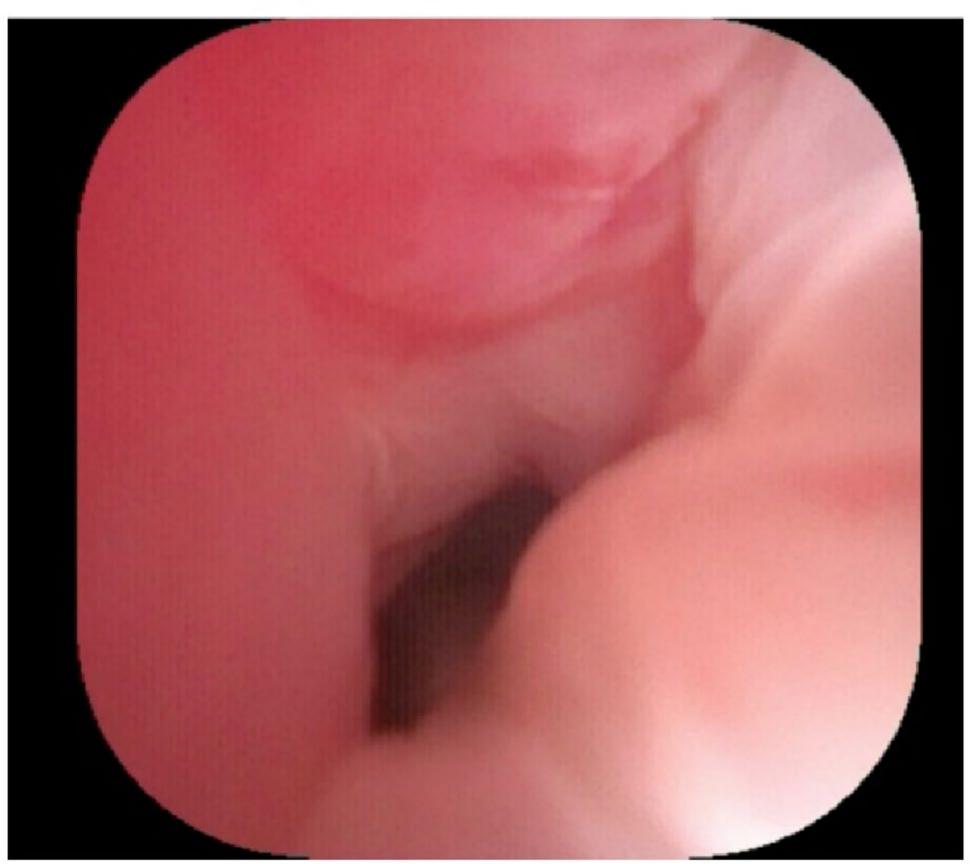
Healthy ureteral mucosa during intraoperative ureteroscopy

**Fig. 5:**
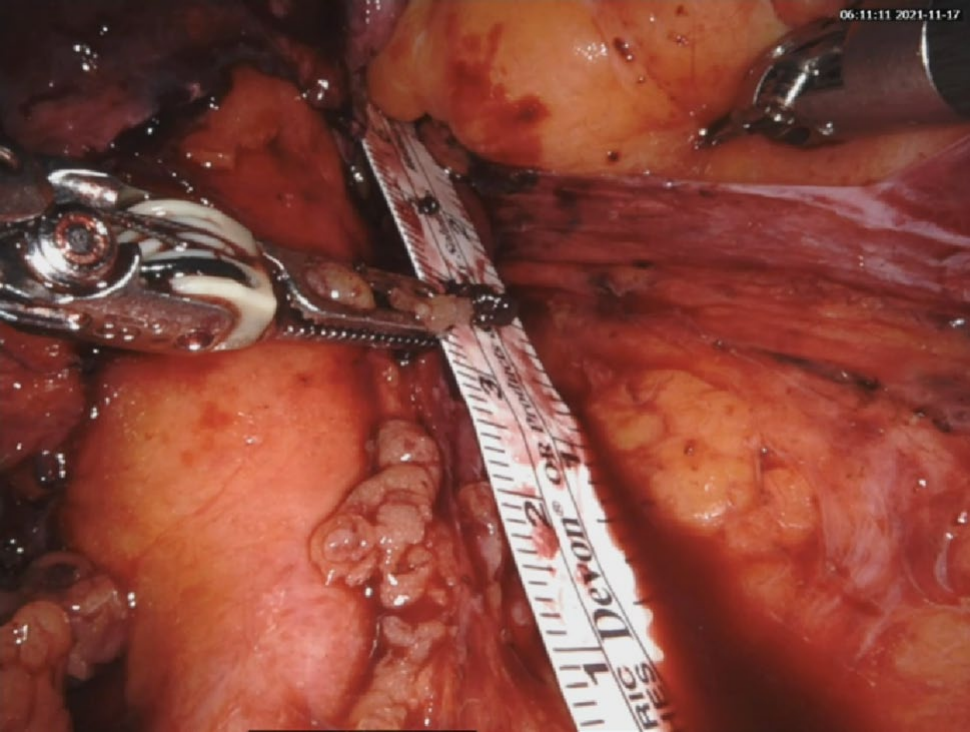
Intraoperative measurement of the stricture length using a sterile ruler

First, ureteroscopy allows precise delineation of the stricture, with direct visualization of its location and length (Figs. 4 and 5). In cases with partial patency, it enables accurate measurement of stricture extent and ensures that no additional strictures are overlooked. Irrigation can further improve visualization by washing the tissue surface.

Second, it is invaluable for intraoperative quality control. Following reconstruction or graft placement, ureteroscopy can confirm can have a widely patent lumen by passing the scope proximally and distally across split-view repair. It also permits removal of concomitant stones, which are frequently present in patients with ureteral stricture disease.

**Fig. 6:**
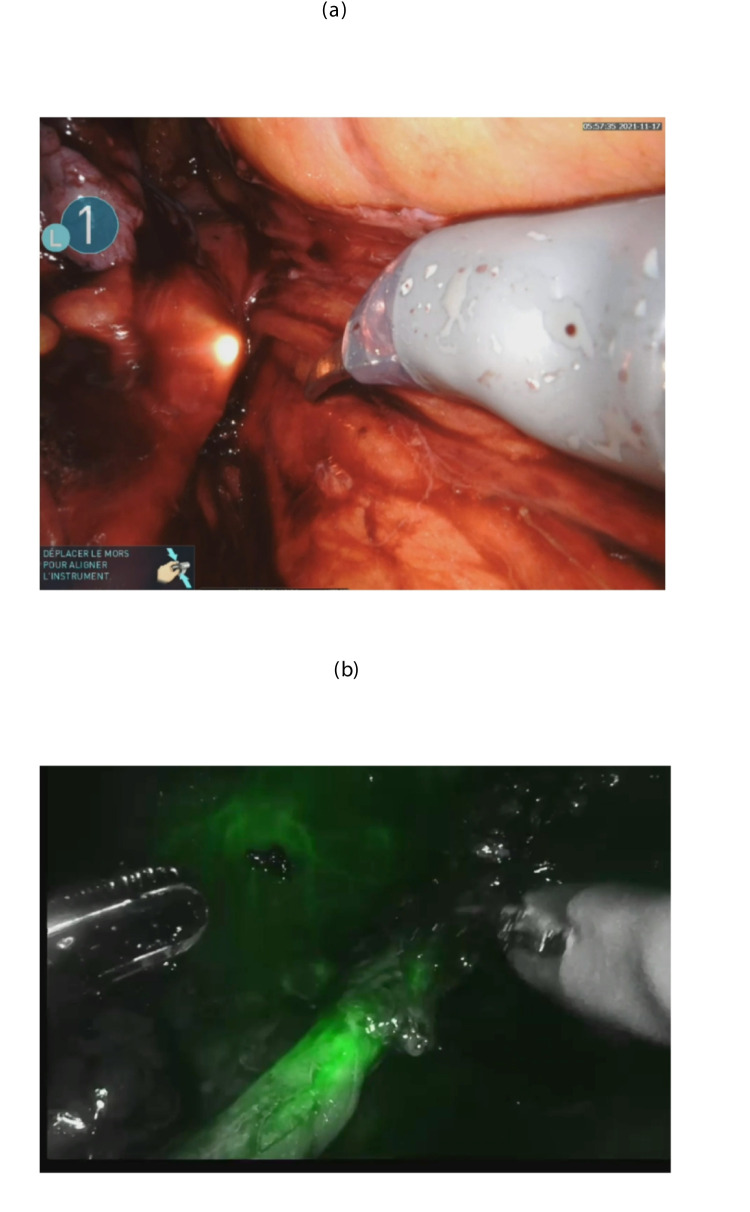
Identification of the ureter intraoperatively with the ureteroscope. **a** Visualization of the illuminated tip of the ureteroscope. **b** Visualization of the illuminated tip of the ureteroscope with the Firefly mode

Third, ureteroscopy is a critical aid in difficult anatomy. When the ureter is obscured or distorted, the ureteroscope provides a reliable guide. Even if the scope itself is not visible, the robotic TilePro® function allows the surgeon to display ureter in addition to the robotic intra-abdominal image (Fig. 4). The Firefly® fluorescence mode can further enhance identification of the ureteroscopic light both the proximal and distal ends of the stricture, which can then be measured (Fig. 5). Additionally, it helps confirm that no other ureteral stricture has been overlooked which can occur sometimes in these cases, especially if the surgeon is in a position where they receive referrals from outside institutions. In some difficult cases, ureteroscopy can also help to locate the ureter when the anatomy is severely modified by using the ‘Firefly’ mode which identifies the light of the ureteroscope (Fig. 6a, b).

Fourth, it can also by using the ‘Firefly’ mode which after reconstructing the stricture to confirm that the repair is sufficiently wide, to test for a watertight anastomosis, and, in some cases, to guide wire placement for a stent.

While the authors strongly recommend the use of intraoperative ureteroscopy when performing RUR, the current literature lacks evidence to support the benefit of intraoperative ureteroscopy in terms of stricture recurrence.

### Other key points in ureteral reconstruction

#### (i) Timing

The timing of ureteral repair has not been evaluated in high-quality studies. A case–control series of 67 patients found no significant difference in recurrence or complications at 15 months between early reconstruction (< 7 days after ureteral injury) and delayed repair [26]. In practice, the decision on when to operate is nuanced and highly dependent on the clinical context. Immediate repair may be feasible if the injury is recognized intraoperatively or in the very early postoperative period, provided that the tissue planes are favorable. In more complex situations—such as difficult dissection, significant inflammation, or poor tissue quality—a period of nephrostomy drainage with elective reconstruction at 6–12 weeks may be more appropriate.

Distal injuries may sometimes be amenable to early repair, particularly when caused by iatrogenic factors such as vaginal surgery, since the intra-abdominal ureter is often unaffected. Conversely, injuries such as thermal damage or postpartum gynecological trauma may present 1–2 weeks later, where deferral is often advisable (e.g., allowing an involuting uterus to settle).

Most proximal strictures present insidiously (e.g. post ureteric stone impaction), making elective reconstruction the preferred approach. Importantly, in patients who have received radiotherapy in the operative field, it may be advisable to defer reconstruction for up to one year after the last dose to allow tissue recovery.

Given the heterogeneity of presentations, there is unlikely to be a universal guideline on optimal timing. A strong element of surgical judgment is required, but in general, postponing reconstruction can be beneficial to allow preoperative optimization and stabilization of the ureteral injury.

#### (ii) Surgical principles, tips and tricks

Successful ureteral reconstruction depends on adherence to fundamental reconstructive principles, with flexibility to adapt in challenging situations:

- Core principles: The cornerstone of repair is joining healthy, well-vascularized tissue in a tension-free anastomosis within a clean field. Although not always fully achievable, these principles remain the foundation of successful outcomes.
- Minimal dissection: Limiting ureteral dissection helps preserve blood supply. In cases of desmoplastic reaction, prior radiotherapy, or dense adhesions, wider dissection may be unavoidable.
- Targeted dissection: Full circumferential mobilization is not always necessary. For example, maintaining the posterior attachment of the ureter, opening it anteriorly, and placing a graft on top can reduce operative time, minimize vascular disruption, and preserve blood flow.
- Renal mobilization (nephropexy): Downward nephropexy is particularly useful when an anastomosis is under tension. Mobilizing the kidney downward and securing the lower pole perinephric fat to the psoas muscle (often with barbed suture) can shorten the gap by up to 5 cm, reducing the tension i.e.ureteroureterostomy with or without augmentation with buccal mucosa graft, Boari flap, ureterocalicostomy [27]. Conversely, upward ureteropexy can be employed by anchoring periureteral tissue near the anastomotic site to similarly reduce tension.

### Overview of the available surgical techniques for RUR

All techniques of surgical repair of ureteral stricture described in open and laparoscopic surgery can be used and have been described in robotic surgery. But a couple of newer surgical techniques have been mostly developed and used with the robotic approach. Below are briefly descriptions of the main techniques of robotic upper urinary tract reconstruction.

#### Ureterolysis

Ureterolysis is undertaken for a variety of indications, the most common being idiopathic retroperitoneal fibrosis (RPF). Other causes include malignant processes that tether the ureter within dense inflammatory and fibrotic tissue, resulting in obstruction. These cases are often complex, frequently involving impaired renal function from longstanding obstruction, or occasionally a solitary functioning kidney.

The principle of ureterolysis is to free the ureter from surrounding pathological tissue, but surgeons must be prepared to apply additional reconstructive techniques if required to restore drainage and preserve function. In some cases, even short strictures are not amenable to ureteroureterostomy due to severe fibrosis limiting mobilization and resulting in too much tension.

For idiopathic RPF, approaches may vary between a supine Trendelenburg or lateral position, though many surgeons favor the lateral position as it allows exposure of the entire ureter—like a nephroureterectomy approach. Dissection is usually begun at a healthy segment of ureter (often near the renal pelvis) and continued distally. The area of maximal involvement is typically recognizable as a zone of dense inflammatory reaction with luminal narrowing, proximal dilation, and then re-entry into normal-caliber ureter.

Traditionally, omental wrapping was considered the standard adjunct following ureterolysis. However, modern practice more commonly favors simple intraperitonization, in which the mobilized ureter is transposed into the peritoneal cavity and separated from the retroperitoneum by a vascularized interposed tissue plane. The omentum remains the preferred option given its abundant bulk, ability to be divided into multiple flaps (particularly useful in bilateral cases), and excellent vascularity. Alternatively, mesenteric or bowel fat can be sutured beneath the ureter to provide separation without the need for circumferential wrapping[28].

#### Ureteroureterostomy

Ureteroureterostomy (UU) is a well-established technique and remains a highly effective option for the management of short ureteral strictures, typically those measuring less than 2 cm. The decision to use UU is usually straightforward, as it requires healthy, well-vascularized tissue and the ability to achieve a tension-free anastomosis.

The standard technique involves spatulating both ureteral ends before performing the anastomosis. Various modifications can be applied in specific situations, such as a side-to-side anastomosis in duplex ureters or a V–Y plasty to gain additional length and reduce tension.

Reported surgical success rates for UU in the literature range from 65 to 100% [3, 29].

#### Ureteroneocystostomy

Ureteral reimplantation into the bladder is performed only for distal strictures. The reimplantation can be refluxing or non-refluxing. When an antireflux mechanism is created, a Lich-Gregoire technique us elected with opening of the detrusor on 4 cm long and opening of the bladder mucosa only on the distal 1 cm of the detrusorotomy. The ureter is spatulated and its distal end is anastomosed to the bladder mucosa. The detrusor is then closed on top of the distal ureter using interrupted Vicryl 2/0 sutures. A Leadbetter-Politano antireflux mechanism can also be used. It is a transvesical technique where the ureter is passed through a supratrigonal, suburothelial tunnel and anastomosed to the mucosa at the distal end of the tunnel. To optimize success for a non-refluxing reimplantation, one should try to adhere to Paquin’s 5:1 tunnel length to ureter diameter ratio. Reported success rates are high, and vary between 85 and 100% in the literature [15, 30, 31].

#### Psoas Hitch

When necessary, a psoas hitch can be performed for ureteral reimplantation to ensure a tension-free anastomosis by mobilizing and securing the bladder to the psoas muscle, frequently with a barbed suture, to spread the tension gradually. Sutures are carefully placed parallel to the length of the psoas muscle and not too deep to minimize the risk of genitofemoral nerve injury. The reimplantation can then be performed in a refluxing or non-refluxing manner. This method typically provides an additional 6 cm of length, and in some cases, up to 10 cm [32]. Accurate stricture recurrence rates are difficult to determine, as most studies do not isolate outcomes from psoas hitch procedures alone[3].

#### Boari flap

This flap is used as an alternative when the psoas hitch does not provide sufficient mobilization. The Boari flap is typically sufficient to bridge a gap of 8 to 15 cm. This technique involves tubularizing a patch of bladder wall, usually measuring approximately 2 × 4 cm [32], or at least a 3:1 length ratio, to create a neo-ureter. The bladder is opened transversally to allow a greater mobilization. It is secured to the psoas muscle as previously described. The bladder it then closed longitudinally to create a tunnel-shaped flap. The anastomosis is subsequently performed between the apex of this flap and the ureter with (terminoterminal) or without (side to side) transection of the later. The anastomosis is usually performed in a refluxing manner. The largest robotic series reported a 90% success rate [33]. A specific complication of the psoas hitch and Boari flap is the potential occurrence of voiding dysfunction, as the reservoir is remodeled. Caution should be used when considering a Boari flap on a radiated bladder, especially in the setting of a high-tension psoas hitch.

#### Side-to-side reimplantation

Side-to-side reimplantation is another option for distal ureteral strictures. In this technique, both the bladder wall and the ureter are incised on their lateral aspects to avoid transecting the ureter. The ureter is then directly anastomosed to the bladder without creating an anti-reflux mechanism. The main drawback of this technique, as for any refluxing reimplantation, is the risk of symptomatic reflux which remains difficult to quantify in the available literature.

#### Buccal mucosa graft ureteroplasty

**Fig. 7:**
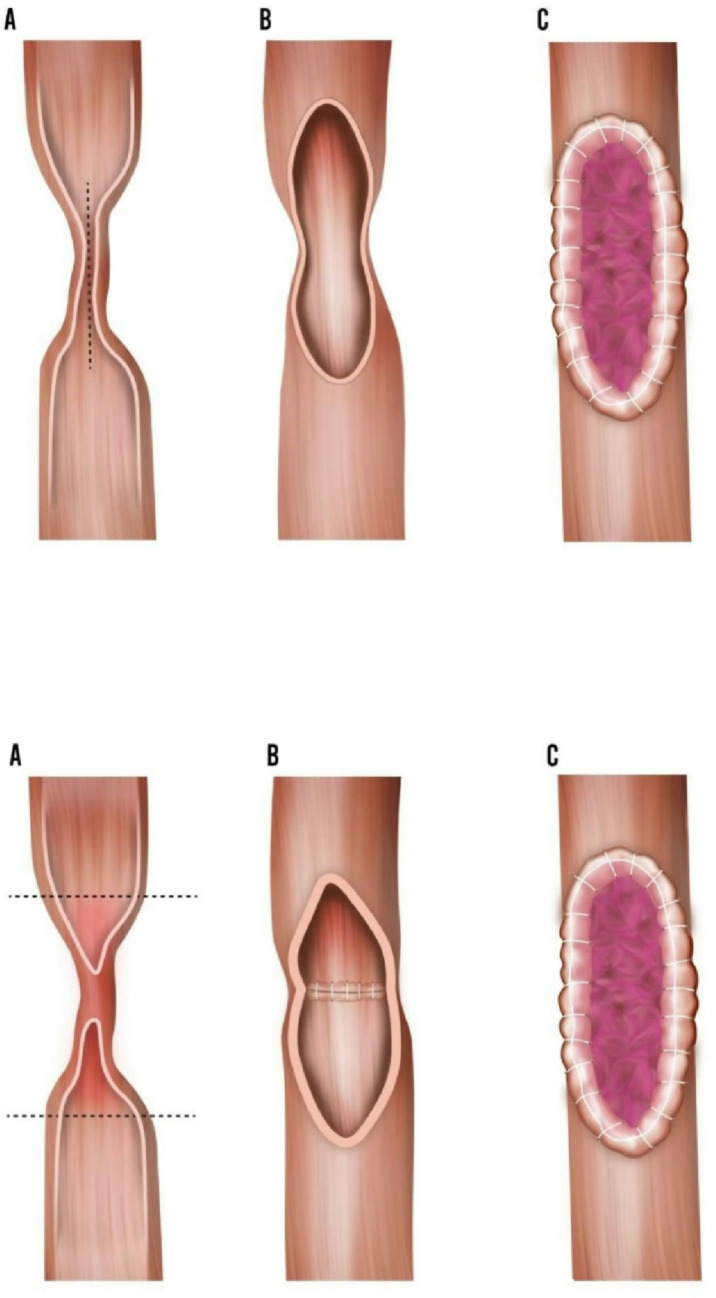
Buccal mucosa graft ureteroplasty techniques. **a** Onlay buccal mucosa graft ureteroplasty technique. **b** Augmented buccal mucosa graft ureteroplasty technique

Buccal mucosa graft has arisen as a revolution in ureteral reconstruction over the past decade. The similarity to ureteral mucosa, along with the ease of harvest and recovery, makes it a highly promising option. Additionally, the use of BMG has been well-documented in urethral reconstruction literature, which cautions against its tubularization due to a recurrence rate of up to 45% [34]. Buccal mucosa can be used in two ways in ureteral reconstruction. The BMG may be placed as an onlay, where the graft is sutured over the opened stricture, and augmented anastomosis, where the graft is applied on the top of the ureteroureterostomy anastomosis after suturing the two healthy ends of the ureter, to decrease the tension (Fig. 7). Supplementing the graft with blood supply, such as omentum, or sewing it onto a well vascularized base such as psoas muscle is highly recommended. Literature reports indicate a high success rate, from 90% up to 100% [35–38].

#### Ureterocalicostomy

For proximal stenosis, a uretero-calicostomy can be performed. This procedure involves connecting the proximal ureter to the lower calyx with the excision of a small portion of the renal parenchyma of the lower pole. This technique is often complemented by a downward nephropexy (see above), mostly useful when the dissection of the renal pelvis is highly challenging. Recurrence rates are likely high but difficult to determine from the literature, as most studies focus on ureteropelvic junction syndromes or redo surgeries [39, 40].

#### Appendix onlay ureteroplasty

Due to its proximity to the ureter, particularly on the right side, as well as its ease of mobilization, the appendix can be useful in ureteral reconstruction. The average length of the appendix, as described in a study of 4,680 specimens, is 8 cm [41]. The appendix should be mostly used as an onlay flap, placed over the incised stricture after being cut longitudinally. The tubularized appendix as a substitution to the strictured portion of the ureter appears to have less success than onlay [42, 43]. It should also be noted that the appendix is not always suitable for use since sometimes the lumen is narrowed or strictured from prior inflammatory episodes. It should also be noted that the appendix is not always suitable for use since sometimes the lumen is narrowed or strictured from prior inflammatory episodes.

#### Ileal ureter

A segment of ileum is harvested and used to replace the ureteral gap. The anastomosis is then performed with the healthy proximal ureter on the proximal side of the ileum, and to the bladder on the distal side of the ileum. Several variations of this technique have been described, such as the reverse-7 procedure, where the two proximal ureters are connected to a long segment of ileum, which is then anastomosed to the bladder wall at the distal ileal end. Overall, ileoplasty for ureteral replacement tends to be associated with higher stricture recurrence rates and complications compared to most other techniques [44, 45].

The literature and current practice support the use of several different surgical techniques for RUR that can be applied in a tailored fashion depending on the clinical presentation.

The definition of surgical success remains highly heterogeneous across published studies, with non-standardized and inconsistently reported criteria. This lack of a uniform, robust outcome definition represents a major limitation and significantly limits the comparability of results across studies.

### Application of surgical technique as per stricture location and length

**Fig. 8:**
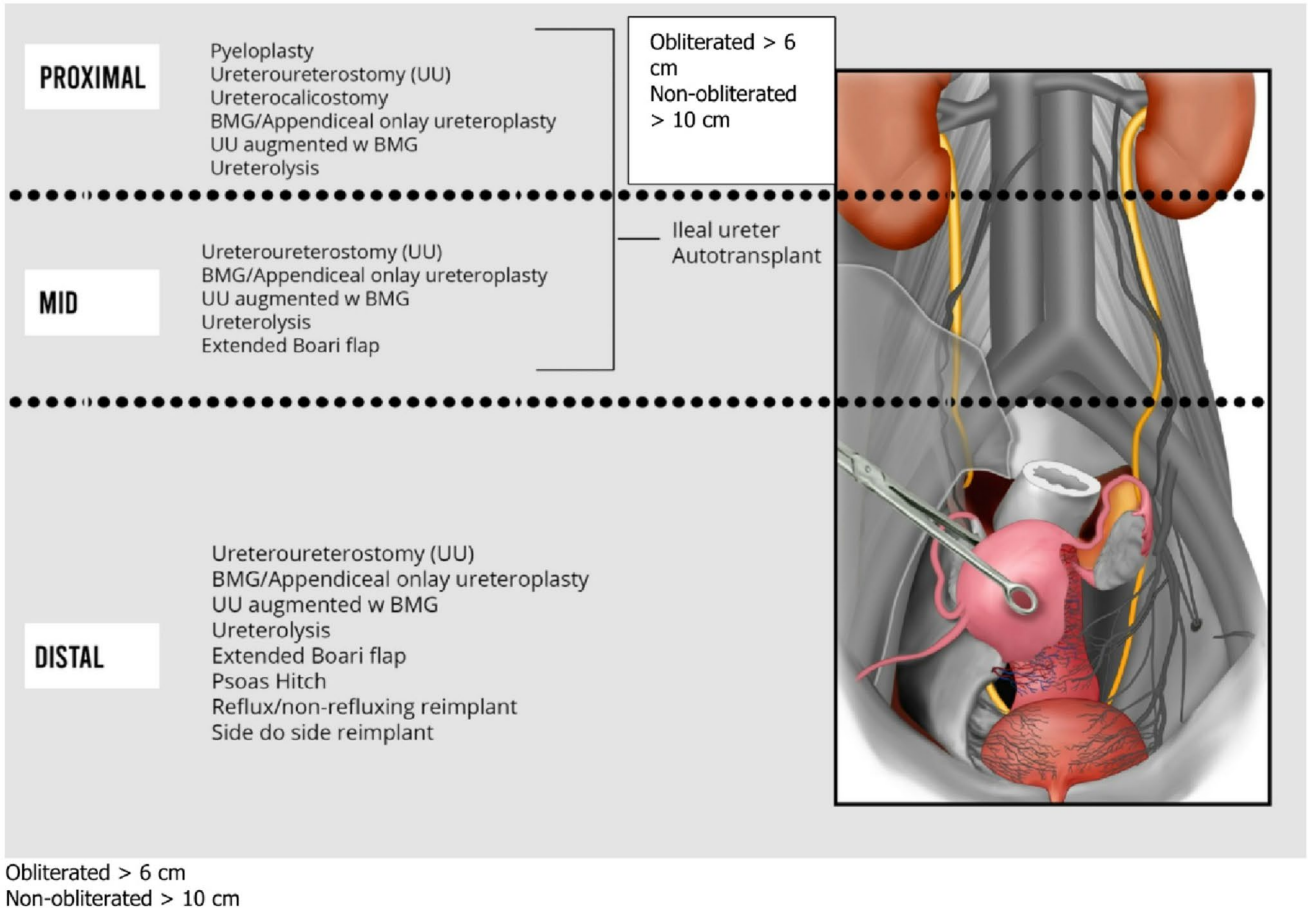
Ureteral reconstruction techniques according to stricture location

**Fig. 9:**
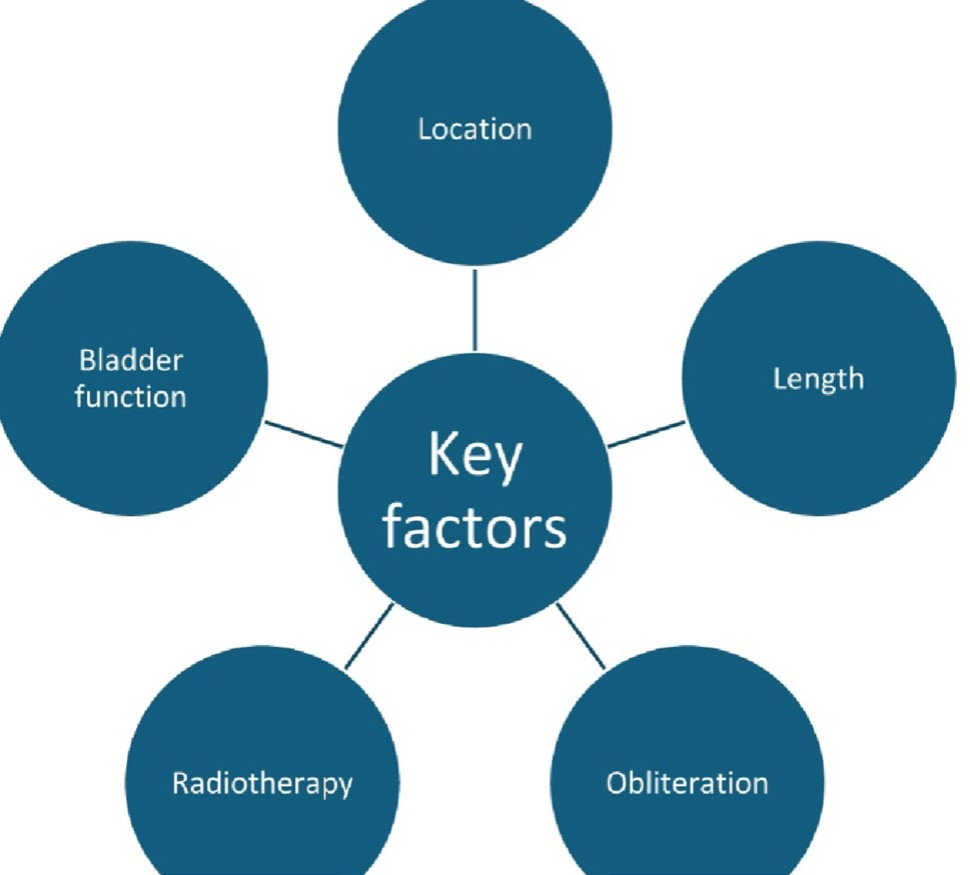
Key decisional factors in ureteral reconstruction

**Fig. 10:**
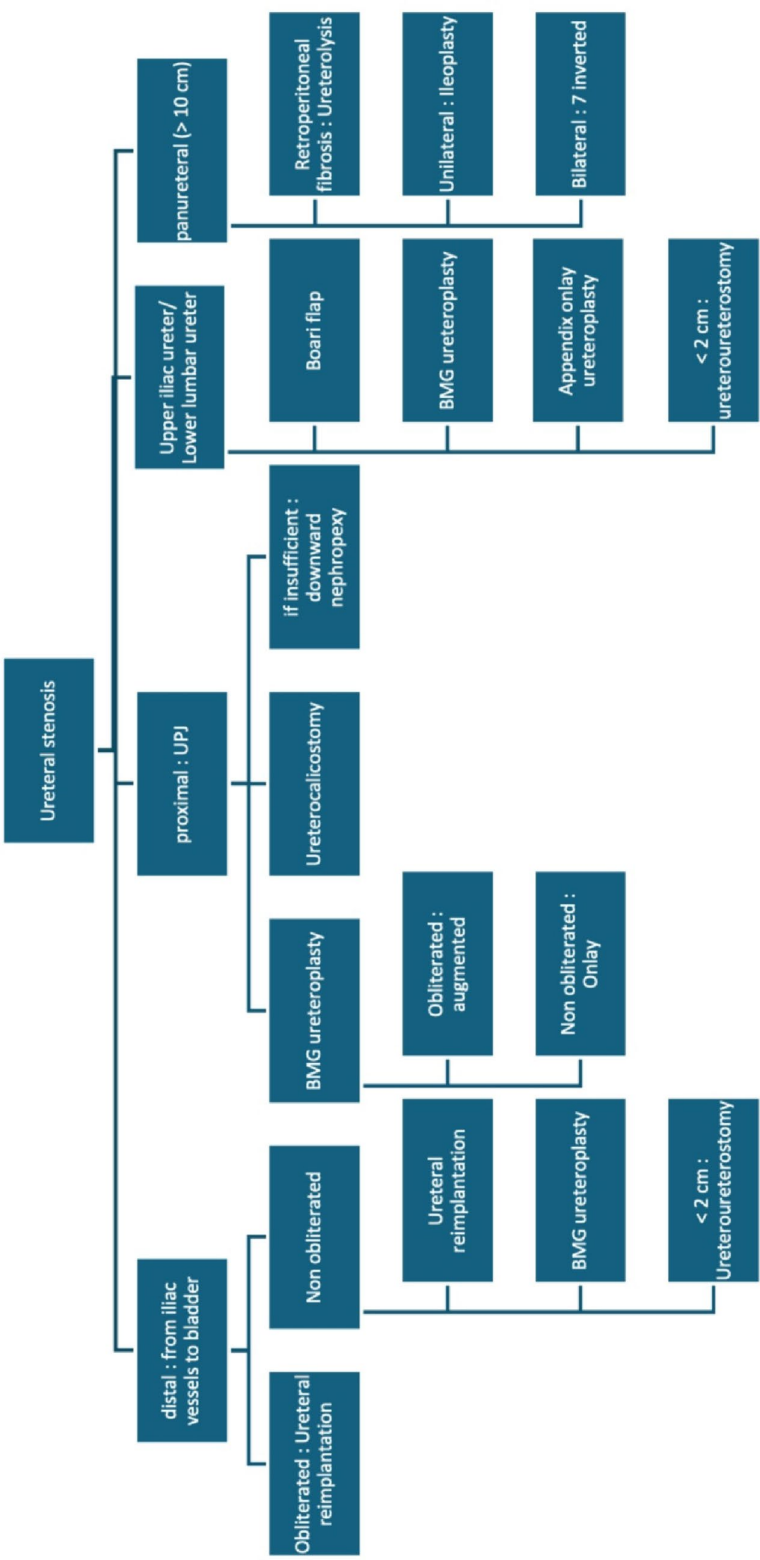
Proposed expert-derived decisional algorithm. *UPJ* Ureteropelvic junction

The application of different available surgical techniques are discussed below based on several key decisional factors: stricture location and length, obliteration, previous radiotherapy, bladder capacity/function (Figs. 8 and 9). A decision-making algorithm is presented in Fig. 10.

#### Management of proximal ureteral stricture

Ureteroureterostomy is often proposed as the first-line approach for strictures less than 2 cm in length. This technique can be applied to all segments of the ureter [46]. A recent review recommends this option for strictures measuring less than 2 cm and located more than 2 cm from the ureterovesical junction, although many favor buccal mucosa graft onlay in those patients as well, to avoid transection of the ureter which may improve its vascularization [29].

Ureterolysis is primarily reported in cases of retroperitoneal fibrosis or endometriosis [47]. In this latter case the viability/blood supply of the ureteral segment should be evaluated, after excision of the endometriosis. If compromised, the impaired segment should be substituted or bypassed. This technique can be used for pan-ureteral narrowing caused by extrinsic factors when a clear stricture location is not identified.

Ureterocalicostomy is used less commonly, with only a few cases reported. It can be an effective option for proximal strictures located close to the ureteropelvic junction, when the renal pelvis is difficult to dissect. Cortical thickness and a low glomerular filtration rate are independent predictors of failure. A portion of renal parenchyma of the lower pole can be excised, to facilitate access to the calix and prevent compression of the anastomosis [48].

Finally, buccal mucosa graft ureteroplasty allows to treat non-obliterated stricture up to 10 cm (onlay) and obliterated stricture up to 6 cm (augmented). Downward nephropexy can also be useful for those locations, to bring the kidney down towards the psoas muscle and decrease the tension on the anastomosis.

#### Management of distal ureteral stricture

Ureteroneocystostomy is one of the most frequently reported techniques and should be considered for strictures located less than 5 cm from the ureterovesical junction [3]. It may lead to acute urinary retention in 8% of cases, particularly in men undergoing prolonged surgery [49]. One recent variation is the side-to-side anastomosis, particularly in cases involving fibrotic tissues that make the dissection challenging. It is also useful in situations where the ureter’s blood supply may be compromised, as it avoids ureteral transection. This technique serves as an alternative to the traditional ureteroneocystostomy [40, 50]. Ureteroneocystostomy can be done in a refluxing or non-refluxing manner.

A psoas hitch can be added to the reimplantation when the available length is insufficient.

The Boari flap is a last resort when the psoas hitch is also inadequate. Various indications for its use are found in the literature. It was traditionally considered for long distal strictures, particularly due to several series showing failure of proximal stenosis repair [51].

Buccal mucosa graft can also be useful in lower ureter stricture, with the significant advantages over reimplantation to preserve the native antireflux mechanism and to avoid bladder mobilization.

#### Suitable techniques for all ureteral stricture

Appendicoplasty is a useful option for right-sided strictures, having been reported for proximal, middle, and even pan-ureteral strictures [52].. No significant difference has been noted between the onlay and interposition techniques, although there is probably a publication bias on that subject, as failed reconstructions are less likely to be reported in the literature[43]. The appendiceal onlay technique has a theoretical advantage over interposition in that it is one longitudinal anastomosis versus two small circular anastomoses. The main disadvantages of this traditional technique are the absence of the appendix in approximately 10–20% of patients and its sometimes insufficient length [53].

Transuretero-ureterostomy is an uncommon procedure, with the primary drawback of potentially compromising the healthy kidney [54].

Buccal mucosa graft ureteroplasty is a versatile option that can be applied to all ureteral segments, using two grafts if necessary. Ileal ureter and renal autotransplantation are also valid but more invasive options.

#### Obliterated vs non obliterated

Obliterated stricture does not allow to use of onlay techniques. Hence, excision-anastomosis techniques are to be favored in these patients. This is also true in non-obliterated strictures when the ureteral wall is of poor quality, for instance when resulting from impacted stones.

#### Radiated vs non radiated

Boari flap or psoas hitch have inferior outcomes in radiated patients[55]. Since the bladder wall may be fibrotic and poorly vascularized due to radiation, bladder mobilization should be avoided whenever possible. Moreover, one should first ensure that bladder function and capacity are sufficient to ensure a decent quality of life after ureteral reconstruction. Otherwise, a urinary diversion should be favored. Hence, a key point is the pre-operative evaluation of lower urinary tract function, with urodynamics and video cystourethrography (or video urodynamics), as it will often unmask severe bladder dysfunctions.

Side to side anastomosis, ileal ureter and buccal mucosa graft ureteroplasties can be useful options in selected radiated patients. The role of older techniques (autotransplantation…) should be reassessed, primarily due to the significant complications they often entail.

Although radiation-induced ureteral strictures represent a particularly challenging subgroup and are associated with poorer outcomes, the current evidence remains limited and heterogeneous, preventing the development of a well-standardized, separate treatment pathway. Therefore, management of irradiated patients should be individualized, taking into account stricture characteristics, tissue quality, previous treatments and overall patient condition.

#### Small bladder capacity/ Bladder dysfunction

In patients with small bladder capacity and/or bladder dysfunction, Boari flap should be avoided, as it may worsen lower urinary tract function. More globally, techniques which do not change bladder anatomy should always be favored when possible.

### What are the good indications of buccal mucosa graft ureteroplasty?

Buccal mucosa graft ureteroplasty is a preferred option in large series of proximal or mid ureteral strictures [40, 56], as well as in complex cases. Both onlay and augmented techniques are reported in the largest case series of 163 patients [35]. Strictures up to 10 cm have been successfully repaired using robotic techniques [36].

Lingual mucosa grafts are very similar to buccal mucosa grafts, with overlapping indications [57, 58]. The difference in complication rates between lingual and buccal mucosa grafts remains controversial [59, 60].

Both grafts can result in harvest site complications in 4% of cases [61]. Graft shrinkage has also been reported in 8–9% of cases [62]. An older series on distal stricture reconstruction reported a low success rate of 60% for open BMG ureteroplasties [63]. However, the success rate is > 90% in almost all existing series [35–37].

In case of non-completely obliterated strictures, it provides a good option while preserving ureteral vascularization, when performing an augmented anastomosis.

### What role left for ileal ureter?

Ileal ureter is a viable option for long or multiple strictures when no other alternatives are available. The largest robotic series reported an average stenosis length of 20 cm [44, 64]. However, complications are frequent, with 8% early and 14% late complications reported in a large open series of 196 cases [65]. Ileal ureter can be a valuable option for patients with a history of radiotherapy or recurrent stricture. A case series also reported excellent outcomes with ileal ureter for complete ureteral avulsion following ureteroscopy [66]. A variation of ileal ureter is the Reverse-7 technique, which is one option for bilateral and extensive strictures, although only a limited number of cases have been described [67].

Many complications have been reported in 11% of large case series of ileoplasties, including ileus, metabolic acidosis in 4% of cases, pyelonephritis, stenosis, and chronic renal dysfunction [17, 68, 69]. Another drawback of this procedure is its numerous contraindications, such as inflammatory bowel disease, enteritis, neurogenic bladder, bladder outlet obstruction and liver or kidney dysfunction [3]. In summary, ileal ureter can be done robotically but should be used only as a last resort option. In summary, ileal ureter can be done robotically but should be used only as a last resort option.

### Is there really still room for kidney autotransplantation?

Autotransplantation has long been considered the gold standard for treating long ureteral strictures. However, vascular complications are common, leading to 4% graft loss and 12% incidence of delayed pseudoaneurysms and chronic loin pain [70]. Only a few robotic cases have been reported. We believe that autotransplantation should be considered only when all other possibilities for restoring renal function have been exhausted.

#### Future directions

Given the heterogeneity and mainly retrospective nature of the currently available evidence, future research in ureteral reconstruction should aim to generate more robust and generalizable data. Randomized controlled trials are unlikely to be feasible due to ethical, logistical, and population size constraints. However, well-designed prospective multicenter registries represent a realistic and highly valuable alternative. Such registries should incorporate standardized definitions of success, consistent reporting of complications, and uniform follow-up protocols to enable meaningful comparison across centers. In addition, collaborative efforts are needed to better define patient selection criteria, refine surgical indications, and evaluate long-term functional outcomes. Ultimately, these initiatives will help establish more reliable evidence and guide clinical decision-making in this evolving field.

## Conclusion

Robot-assisted ureteral reconstruction offers numerous advantages and has significantly shifted the paradigm in the management of ureteral strictures. Buccal mucosa graft ureteroplasty appears to be a highly promising option, demonstrating both effectiveness and safety, and may potentially emerge as a preferred approach in selected patients as further evidence accumulates. Its use can be expanded beyond short proximal strictures, to include longer strictures up to 10 cm and distal strictures. Several key surgical principles have been put forward over the past few years such as ureteral rest, intraoperative ureteroscopy or indocyanine green use. In the absence of robust evidence underpinning current practice, this expert review presents a practical algorithm and key surgical techniques to guide clinical practice. Future work should focus on standardizing practice and outcomes where possible to allow pooling of data and improving the evidence guiding our practice.

## Figures and Tables

**Annex 1:**
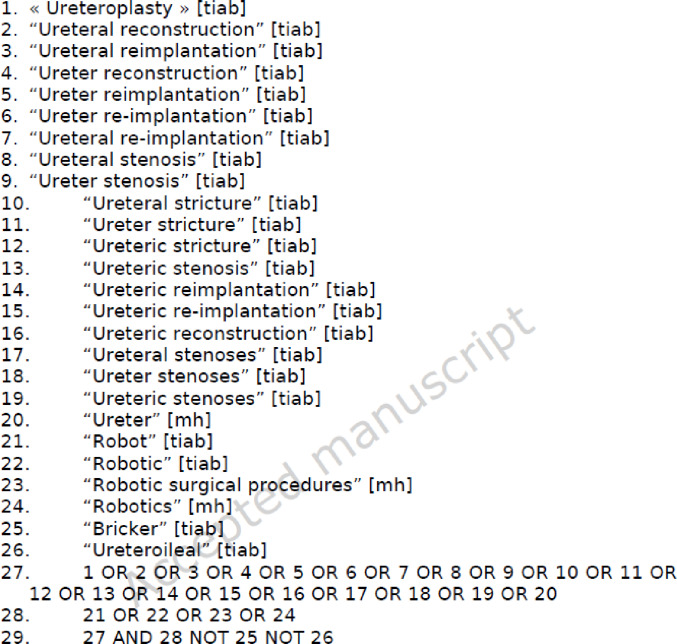
Search algorithm

## Appendix: Pubmed and Cochrane

See Annex 1.
