# Correction: Ureteral stricture: current treatment algorithm and key surgical principles in the robotic upper urinary tract reconstruction era

**DOI:** 10.1007/s00345-026-06328-x

**Published:** 2026-04-11

**Authors:** Alice Bourillon, Benoit Peyronnet, Barry B. McGuire, Ugo Pinar, Ziho Lee, Rajesh Nair, Michael Stifelman, Daniel Eun, Lee C. Zhao, Nikita Bhatt

**Affiliations:** 1 Department of Urology, University Hospital of Rennes, 2 Rue Henri Le Guilloux, Rennes, France; 2 Department of Urology, St Vincent’s University Hospital, Dublin, Ireland; 3 Department of Urology, La Pitié Salpêtrière Hospital, Sorbonne University, Paris, France; 4 Department of Urology, Northwestern University Feinberg School of Medicine, Chicago, IL USA; 5 Department of Urology, Guy’s Hospital, London, UK; 6 Department of Urology, Hackensack Meridian School of Medicine, Nutley, NJ USA; 7 Department of Urology, Lewis Katz School of Medicine at Temple University, Philadelphia, PA USA; 8 Department of Urology, NYU Langone Health, New York, USA

**Correction: World Journal of Urology (2026) 44:102**

10.1007/s00345-025-06181-4

In the original version of the article, the following author name & affiliation has been missed out,

Author: Nikita Bhatt

Affiliation: St Vincent’s University Hospital, Dublin

The original article has been updated.

